# Laparoscopic surgery for colorectal cancer with persistent descending mesocolon

**DOI:** 10.1186/s12957-019-1734-1

**Published:** 2019-11-11

**Authors:** Yukiharu Hiyoshi, Yuji Miyamoto, Kojiro Eto, Yohei Nagai, Masaaki Iwatsuki, Shiro Iwagami, Yoshifumi Baba, Naoya Yoshida, Hideo Baba

**Affiliations:** Department of Gastroenterological Surgery, Graduate School of Medical Sciences, Kumamoto University, 1-1-1 Honjo, Kumamoto, 860-8556 Japan

**Keywords:** Colorectal cancer, Laparoscopic surgery, Persistent descending mesocolon, IR

## Abstract

**Background:**

Persistent descending mesocolon (PDM) is caused by the absence of fusion of the descending colon to the retroperitoneum. We herein report two colorectal cancer cases with PDM that were treated with laparoscopic surgery.

**Case presentation:**

Case 1: a 50-year-old man with sigmoid colon cancer and synchronous liver metastasis. After neoadjuvant chemotherapy, he underwent laparoscopic sigmoidectomy with lymph node dissection cutting the root of the inferior mesenteric artery (IMA) and synchronous liver resection. He experienced postoperative stenosis of the reconstructed colon possibly due to an impaired arterial blood flow in the reconstructed colon.

Case 2: a 77-year-old man with rectal cancer. Laparoscopic low anterior resection preserving the left colic artery (LCA) was performed. Intraoperative infrared ray (IR) imaging using indocyanine green (ICG) showed good blood flow of the reconstructed colon. He had no postoperative complications. In cases of PDM, the mesentery of the descending and sigmoid colon containing the LCA is often shortened, and the marginal artery of the reconstructed colon is located close to the root of the LCA. Lymph node dissection accompanied by cutting the LCA carries a risk of marginal artery injury. Therefore, we recommend lymph node dissection preserving the LCA in colorectal cancer patients with PDM in order to maintain the blood flow of the reconstructed colon. If the IMA and LCA absolutely need to be cut for complete lymph node dissection, the marginal artery should be clearly identified and preserved. In addition, intraoperative IR imaging is extremely useful for evaluating colonic perfusion and reducing the risk of anastomotic complications.

**Conclusion:**

In colorectal cancer surgery in patients with PDM, surgeons should be aware of these tips for maintaining the blood flow of the reconstructed colon and thereby avoid postoperative complications caused by an impaired blood flow.

## Background

The descending mesocolon normally is fused to the posterior and lateral parietal peritoneum by the end of the fifth month of gestation [1]. Persistent descending mesocolon (PDM) is a developmental anomaly characterized by the colonic mesentery’s failure to fuse with the dorsal abdominal wall [1]. In patients with PDM, the descending colon may be shifted to a more medial position than usual, and the sigmoid colon is located at the right side of the abdomen; therefore, PDM is sometimes called “right-sided sigmoid colon” [2, 3]. Although some clinical complications of PDM, such as primary intestinal obstruction, colonic volvulus, and intussusception, have been reported [4–6], this condition is asymptomatic in most cases. In addition, in certain cases undergoing colon cancer surgery, characteristic anomalies, including adhesion between the mesentery and pelvic wall and a short descending mesentery, as well as the importance of careful surgical procedures have been described [7, 8].

We herein report two patients with PDM who underwent laparoscopic surgery for colorectal cancer. The first case experienced postoperative stenosis of colonic anastomosis and it seemed to be caused by impaired blood flow of the reconstructed colon. Therefore, in the second case, we tried to maintain a good blood flow of the reconstructed colon to avoid anastomotic complications.

## Case presentation

### Case 1

**Fig. 1:**
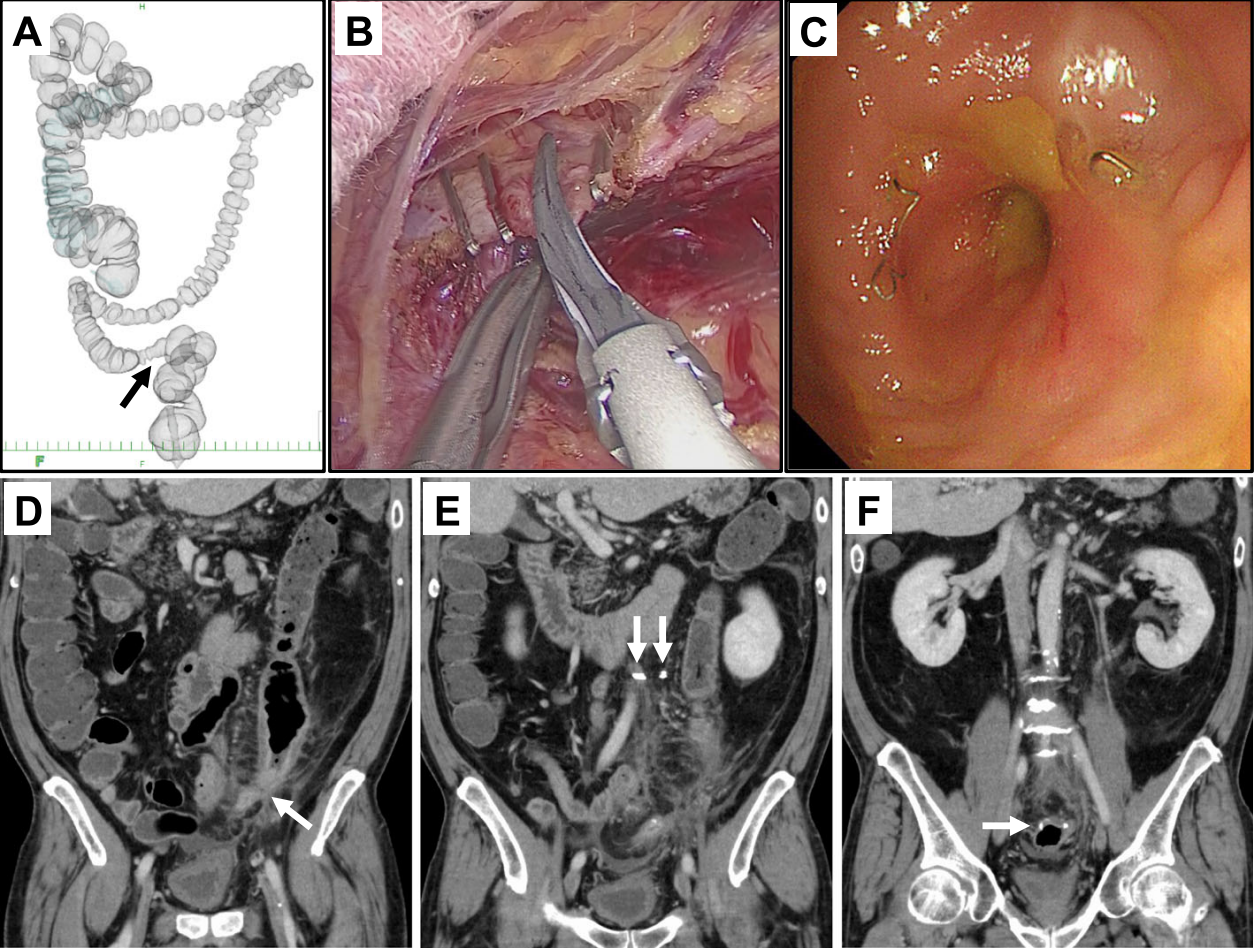
Case 1: a case of upper rectal cancer with postoperative stenosis of the reconstructed colon. **a** Preoperative computed tomography (CT)-colonography shows the PDM. The black arrow shows sigmoid colon cancer. **b** During laparoscopic surgery, the root of the inferior mesenteric artery (IMA) was cut. **c** Colonoscopy shows postoperative stenosis of the reconstructed. **d**–**f** CT shows colonic dilatation caused by stenosis of the reconstructed colon. **d** The white arrow shows colonic stenosis and dilatation of the oral side. **e** The white arrows show the clips on the IMA (left) and left colic artery (LCA) (right). The contrast enhancement at the distal side of the reconstructed colon was weak. **f** The white arrow shows the staple line at the anastomosis

A 50-year-old man presented with a 2-month history of melena. Colonoscopy and computed tomography (CT) showed sigmoid colon cancer and synchronous liver metastasis. CT-colonography showed PDM and a primary tumor at the distal side of the sigmoid colon (Fig. 1a). After systemic chemotherapy (Panitumumab and FOLFOX), he underwent laparoscopic sigmoidectomy with the double-stapling technique (DST) anastomosis and synchronous liver resection. The sigmoid colon located at the right side of the abdomen adhered to the abdominal wall, and the adhesion was dissected laparoscopically. In this case, the root of the inferior mesenteric artery (IMA) was cut for lymph node dissection (Fig. 1b). The left colonic artery (LCA) and inferior mesenteric vein (IMV) next to the IMA were then also cut. The operation time was 835 min and the volume of bleeding was 285 ml. Two months after surgery, the patient developed abdominal distension and pain. Colonoscopy showed stenosis of the reconstructed colon (Fig. 1c), and CT showed colonic dilatation caused by stenosis at the oral side of the anastomosis (Fig. 1d–f). No findings of local recurrence were seen. The clip on the LCA was located close to the marginal artery (Fig. 1e), and contrast enhancement at the distal side of the reconstructed colon was weak. Therefore, the main cause of the stenosis seemed to be an impaired arterial blood flow of the reconstructed colon. Although the patient was admitted to our hospital and fasted, his complaint and colonic dilatation prolonged. Because he had obstructive colitis caused by severe colonic stenosis, he underwent colostomy at the transverse colon to improve the colonic dilatation 4 months after colectomy. One year after primary surgery, he experienced recurrence in his liver. He underwent second liver resection and has been treated with systemic chemotherapy, and is still alive for 24 months after primary surgery.

### Case 2

**Fig. 2:**
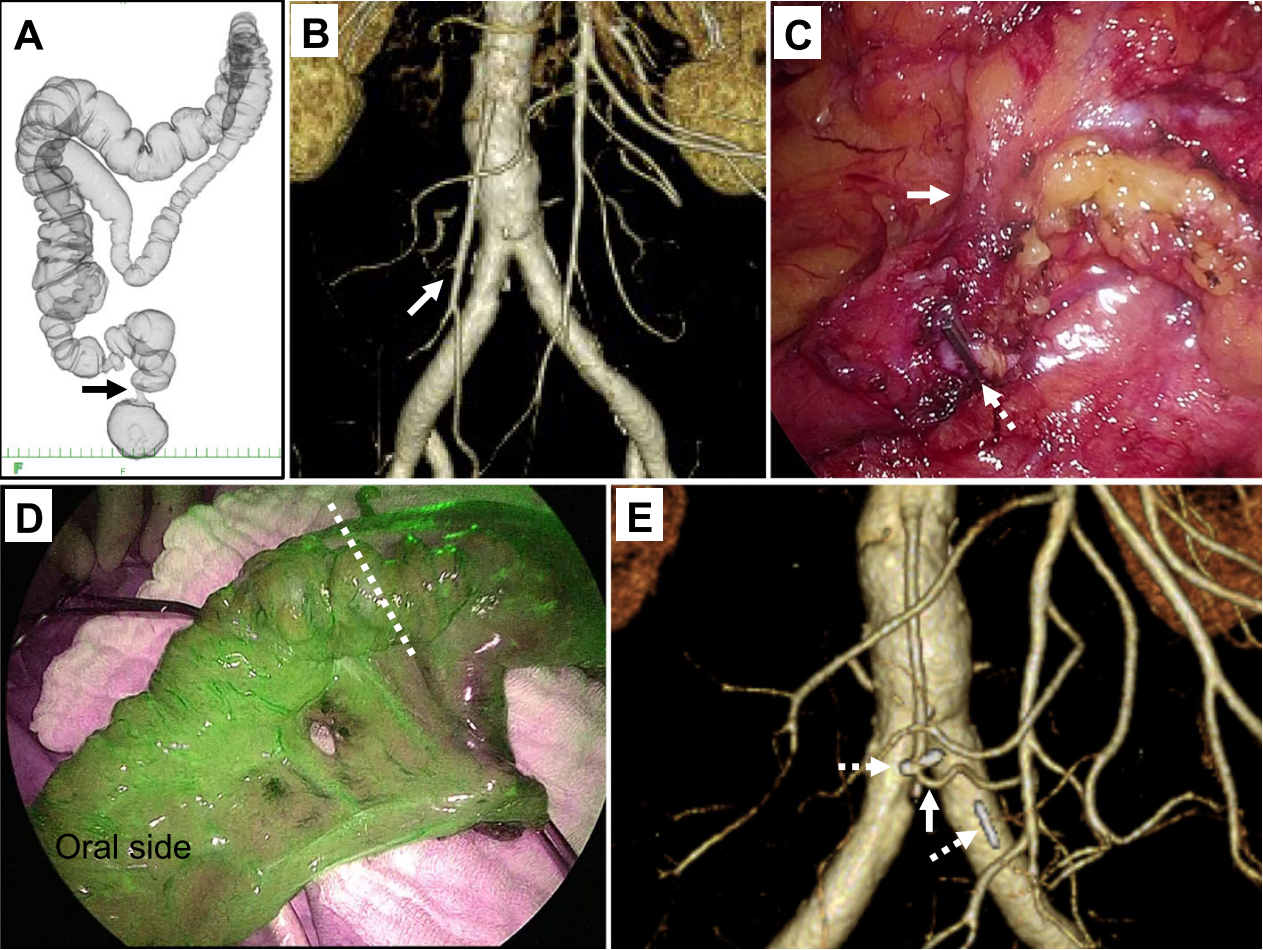
Case 2: a case of middle rectal cancer without any postoperative complications. **a** Preoperative computed tomography (CT)-colonography shows the PDM. The black arrow shows middle rectal cancer. **b** Preoperative CT-angiography. The white arrow shows that the left colonic artery (LCA) branched from the inferior mesenteric artery (IMA). **c** During laparoscopic surgery, the superior rectal artery (SRA) was cut (white dotted arrow) while preserving the LCA (white arrow). **d** Infrared ray (IR) imaging using indocyanine green (ICG) performed prior to the anastomosis. The picture was taken after the rectal transection of the anal side of the tumor. After the transection, oral side of the colon with the tumor was pulled out from the umbilicus, and ICG (3 ml, 7.5 mg) was injected. ICG fluorescence imaging using a near-infrared camera system showed good blood flow of the reconstructing colon at the estimated cut line (white dotted line). **e** Postoperative CT-angiography. The white arrow shows the preserved LCA. The white dotted arrows show the clips on the SRA (left) and inferior mesenteric vein (IMV) (right)

A 77-year-old man who had difficulty defecating was diagnosed with rectal cancer by colonoscopy. CT-colonography showed PDM and a primary tumor at the middle rectum (Fig. 2a). Preoperative CT-angiography showed that the LCA branched from the IMA (Fig. 2b). He underwent laparoscopic low anterior resection with D3 lymph node dissection and the DST anastomosis preserving pelvic autonomic nerves. The sigmoid colon located at the right side of the abdomen adhered to the abdominal wall. In addition, the mesocolon and the mesentery of the ileum were adhered. These adhesions were dissected initially. In laparoscopic low anterior resection, the superior rectal artery (SRA) was cut while preserving the LCA (Fig. 2c). Intraoperative infrared ray (IR) imaging using indocyanine green (ICG) performed prior to the DST anastomosis showed good blood flow of the reconstructed colon (Fig. 2d). The anastomosis was located at 5 cm from the anal verge. The operation time was 333 min and the volume of bleeding was 10 ml. The preserved LCA was identified on postoperative CT-angiography (Fig. 2e). He had no postoperative complications. Pathological examination showed pT3N2bM0 pStage IIIC (TNM classification eighth edition) with a negative resection margin. He experienced an early recurrence in his liver and lung 1 month after surgery and has been treated with oxaliplatin-based systemic chemotherapy without any anastomotic complication. He is still alive for 13 months after surgery.

## Discussion and conclusions

To our knowledge, there are few reports regarding laparoscopic surgery for colorectal cancer in patients with PDM [7, 8]. In this report, we described two cases of PDM treated with laparoscopic surgery for colorectal cancer. Case 1 experienced postoperative colonic stenosis near the anastomosis mainly due to an impaired arterial blood flow of the reconstructed colon. Ischemia at the anastomosis had been described as a possible risk factor of anastomotic stenosis [9]. In contrast, case 2 had no postoperative complications thanks to the following efforts to maintain a good blood flow of the reconstructed colon: (1) the evaluation of the colonic arterial supply by preoperative computed tomography-angiography, (2) confirmation of complete adhesiolysis and mobilization of sigmoid colon, (3) making the appropriate arterial cut in lymph node dissection, and (4) performing intraoperative IR imaging using ICG.

**Fig. 3:**
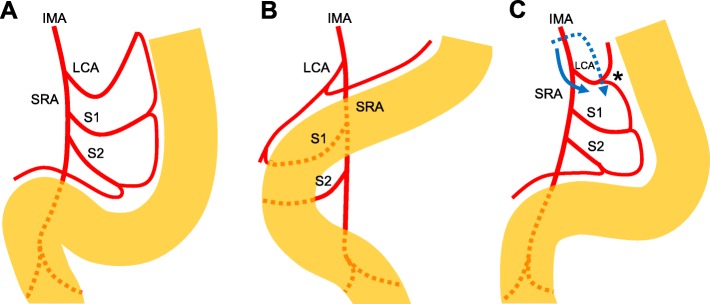
The anomalous branching pattern of the inferior mesenteric artery (IMA) supplying the PDM. **a** The IMA supplying the normal sigmoid colon. **b** The IMA supplying the descending and sigmoid colon with PDM. **c** The IMA supplying the surgically mobilized descending and sigmoid colon. Because the mesentery of the colon containing the LCA is shortened, the cut LCA (shown by the blue dotted line) has a risk of injury of the marginal artery (*). In contrast, the blue line shows the SRA cut preserving LCA which can avoid injury of the marginal artery. IMA, inferior mesenteric artery; LCA, left colonic artery; SRA, superior rectal artery; S, sigmoid colonic artery

In cases of PDM, the sigmoid colon and mesocolon often adhere to the abdominal cavity [2, 3, 7, 8] and such adhesion was seen in both cases we presented. In addition, compared with the normal sigmoid colon (Fig. 3a), the descending colon is located toward the medial side and the sigmoid colon is located at the right side of the abdomen (Fig. 3b). After completing adhesiolysis and mobilizing the sigmoid colon to the normal position, the mesocolon containing the LCA consequently becomes shortened (Fig. 3c) as previously reported [2]. Importantly, the fact that the descending colon artery, sigmoid colon artery, and superior rectal artery often branch radially from the IMA should have been noted during the operation [7]. Therefore, if the IMA and LCA are cut for lymph node dissection (Fig. 3c, blue dotted line), there is a risk of injuring the marginal artery. However, cutting the SRA while preserving the LCA (Fig. 3c, blue line) can avoid such potential injury. In case 1, the marginal artery of the reconstructed colon might have been injured, as the clip on the LCA was located close to the marginal artery (Fig. 1e). Therefore, we recommend lymph node dissection preserving the LCA in colorectal cancer surgery in patients with PDM. If the IMA and LCA absolutely need to be cut for complete lymph node dissection, the marginal artery should be clearly identified and preserved after complete adhesiolysis and mobilization of the descending colon and the sigmoid colon to the normal position.

Recent reports have shown that IR imaging is extremely useful for evaluating colonic perfusion and reducing the risk of anastomotic complications [10, 11]. We ensured a good arterial blood flow of the reconstructed colon using intraoperative IR imaging and believe that this technique is extremely useful. If the blood flow of the reconstructed colon is poor, additional resection of the colon may be needed. In the present case 2, the LCA was preserved in order to avoid injuring the marginal artery, and intraoperative IR imaging was performed in order to confirm the arterial blood flow, just in case. However, we believe that the evaluation of the blood flow is useful, especially in cases of PDM with the root of the IMA cut off for lymph node dissection.

We described the tips and pitfalls associated with colorectal cancer surgery in patients with PDM. Particularly when treating patients with descending colon cancer, sigmoid colon cancer, and rectal cancer, surgeons should recognize these tips for ensuring good blood flow to the reconstructed colon and thereby avoid postoperative complications caused by an impaired blood flow.

## Data Availability

Not applicable
